# Radiogenomics and Radiomics in Liver Cancers

**DOI:** 10.3390/diagnostics9010004

**Published:** 2018-12-27

**Authors:** Aman Saini, Ilana Breen, Yash Pershad, Sailendra Naidu, M. Grace Knuttinen, Sadeer Alzubaidi, Rahul Sheth, Hassan Albadawi, Malia Kuo, Rahmi Oklu

**Affiliations:** 1 Division of Vascular and Interventional Radiology, Minimally Invasive Therapeutics Laboratory, Mayo Clinic, Phoenix, AZ 85054, USA; Saini.Aman@mayo.edu (A.S.); Breen.Ilana@mayo.edu (I.B.); ypershad@stanford.edu (Y.P.); Naidu.Sailen@mayo.edu (S.N.); knuttinen.grace@mayo.edu (M.G.K.); alzubaidi.sadeer@mayo.edu (S.A.); albadawi.hassan@mayo.edu (H.A.); malia1274@gmail.com (M.K.); 2 Department of Interventional Radiology, MD Anderson Cancer Center, Houston, TX 77030, USA; RASheth@mdanderson.org

**Keywords:** radiogenomics, radiomics, hepatocellular carcinoma, intrahepatic cholangiocarcinoma, liver metastasis

## Abstract

Radiogenomics is a computational discipline that identifies correlations between cross-sectional imaging features and tissue-based molecular data. These imaging phenotypic correlations can then potentially be used to longitudinally and non-invasively predict a tumor’s molecular profile. A different, but related field termed radiomics examines the extraction of quantitative data from imaging data and the subsequent combination of these data with clinical information in an attempt to provide prognostic information and guide clinical decision making. Together, these fields represent the evolution of biomedical imaging from a descriptive, qualitative specialty to a predictive, quantitative discipline. It is anticipated that radiomics and radiogenomics will not only identify pathologic processes, but also unveil their underlying pathophysiological mechanisms through clinical imaging alone. Here, we review recent studies on radiogenomics and radiomics in liver cancers, including hepatocellular carcinoma, intrahepatic cholangiocarcinoma, and metastases to the liver.

## 1. Introduction

Primary liver cancers, including hepatocellular carcinoma (HCC) and intrahepatic cholangiocarcinoma (ICC), are a major cause of cancer-related mortality worldwide. In 2018 alone, there were 841,080 new cases of liver cancers and 781,631 deaths globally [1]. As with any malignancy, earlier detection portends a better prognosis, and for HCC (the most common primary liver cancer), 5-year survival rates are excellent for tumors under 2 cm, while 5-year survival for larger tumors can drop to under 10% [2]. Intrahepatic cholangiocarcinoma (ICC), the second most common primary liver cancer, follows similar survival trends, underscoring the importance of early detection and intervention.

**Figure 1:**
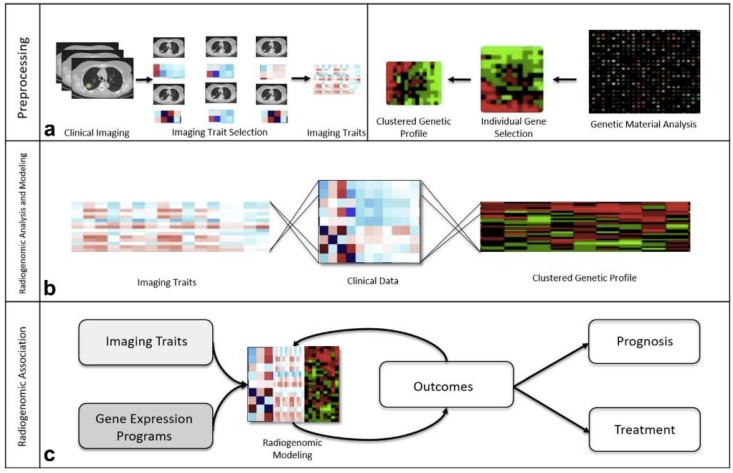
Diagram of the radiogenomic process. (**a**) First, imaging traits or “radiographic phenotypes”, such as tumor shape, intensity, margins, and texture, are extracted from segmented images. Genomic maps indicating the expression of individual genes or gene clusters are created from the given tissue specimen. Then, the imaging traits, genomic maps, and other clinical data, including histopathologic information or tumor marker data, are statistically analyzed and aggregated to form a radiogenomic association map. (**b**) The radiogenomic model is used to gain clinical data, including prognostic and predictive information, for a given set of imaging features and genomic information. (**c**) Incorporation of outcomes data into the radiogenomic model leads to further refinement and model validation. Reproduced with permission from [4], published by Elsevier, 2018.

Tumor biology is being increasingly recognized as an important driver of prognosis and outcomes. Over the past 20 years, advances in the field of genomics have greatly increased our ability to understand tumor behavior and personalize therapeutic approaches. Similar advances in medical imaging techniques and analysis have allowed radiologists to see beyond tumor morphology and extract and correlate quantitative and qualitative data from cross-sectional imaging with genetic information (Figure 1). This rapidly developing field, termed radiogenomics, has shown great promise for a number of different malignancies and represents the “evolution of radiology-pathology correlation from the anatomical-histological level to the genetic level” [3]. This emerging field should be differentiated from the field of radiation genomics, which attempts to study the role of genetics in response to radiation therapy and is not the focus of this paper. The term, radiogenomics, is often used to describe both fields. The field of radiomics centers on the extraction and analysis of quantitative imaging features in an attempt to improve clinical decision making by providing diagnostic, prognostic, and predictive data in a non-invasive and repeatable fashion. Taken together, both radiogenomics and radiomics hold great potential for personalized cancer care. Here, we will examine radiogenomics and radiomics studies to date involving cancers of the liver, including HCC, ICC, and also metastatic disease. 

## 2. Discussion

### 2.1. Radiogenomics in Liver Cancers

To date, there are a limited number of studies on radiogenomics in neoplasms affecting the liver. In 2007, Segal and colleagues were the first to investigate the correlation between an HCC tumor’s gene expression pattern and its imaging features. In a study of 28 patients with HCC, the researchers identified 32 imaging traits from three-phase contrast enhanced computed tomography (CT) scans and then correlated these traits to the expression levels of 116 genetic markers selected from a total of 6732 genes determined by microarray analysis [5]. The resulting association map, correlating these imaging features to gene expression levels, was statistically validated in an independent set of tumors. The investigators noted that, on average, only three imaging traits were needed to capture the variation in expression of any particular gene marker, and more importantly, combinations of 28 imaging traits could account for the variation of all 116 gene markers [5]. Furthermore, the genes in specific molecular profiles shared common physiologic functions, such as cell proliferation or liver enzyme synthesis, and these functions could be correlated to specific imaging features [5]. Finally, the researchers were able to extract prognostic information from their association map through the identification of two imaging traits—“the presence of internal arteries” and “absence of hypodense halos”—that were associated with a previously identified “venous invasion signature,” an imaging pattern that has previously been shown to predict microscopic venous invasion (MVI) and overall survival (OS) [5]. The findings of this study suggest the possibility of a fast, non-invasive method for obtaining the genetic profile of an HCC tumor. This information, combined with histopathologic data, could be also be used for prognostic and therapeutic stratification as well.

In the same year, Kuo et al. performed a radiogenomic analysis to identify imaging features in HCC associated with a gene expression profile composed of 61 previously identified genes that indicated tumoral responsiveness to doxorubicin. The contrast-enhanced CT’s of 30 HCC tumors were analyzed for six imaging features that were then correlated to microarray data on approximately 18,000 genes [6]. An imaging feature known as “tumor margins on arterial phase images” was found to be significantly associated with doxorubicin response [6]. Specifically, higher tumor margins on arterial phase scores were associated with increased venous invasion and clinical stage—both negative prognostic factors in HCC. Furthermore, an imaging trait known as “internal arteries” was found to be associated with the expression program of genes involved with the synthetic function of normal liver and the presence of “internal arteries” was associated with poor differentiation of tumor cells and venous invasion [6].

The use of imaging features or radiogenomics biomarkers to predict MVI was further studied by Banerjee et al., in a multicenter, prospective cohort of 157 HCC patients prior to surgical resection or transplant. Through the correlation of the expression levels of the 91 gene venous invasion panel with three imaging features, including “internal arteries”, “hypodense halos”, and “tumor-liver difference”, on contrast-enhanced CT, the researchers measured a radiogenomics venous invasion score and determined its diagnostic accuracy. For the entire patient cohort, the diagnostic accuracy, sensitivity, and specificity were found to be 89%, 76%, and 94%, respectively. Furthermore, those with higher scores for radiogenomic venous invasion were found to have worse OS and 3-year local recurrence, compared to patients with a lower score [7]. These results highlight the prognostic ability and clinical utility of radiogenomics analysis. By identifying patients with a higher likelihood of recurrence, alternative treatments and follow up approaches can be considered.

Renzulli et al. also investigated the use of radiogenomics in predicting MVI in HCC through a retrospective study of 125 pre-operative patients. In addition to the imaging features used by Banerjee et al., Renzulli and colleagues also included “maximum diameter”, “number of lesions”, and “peritumoral enhancement”. The researchers found that, in addition to “hypodense halos” and “internal arteries”, “large tumor size”, “non-smooth margins”, and “peritumoral enhancement” were significantly associated with the presence of MVI, while “number of lesions” was not [8]. This study validated the results of previous studies, confirming that certain imaging features in HCC can be associated with angiogenesis, cellular proliferation, and matrix invasion [5]. In a paper by Taouli et al., imaging features of HCC were correlated to predictive genetic signatures in 38 pre-operative patients. The investigators found strong associations between imaging features, such as “infiltrative pattern”, “mosaic appearance”, and “presence of macrovascular invasion”, and previously identified aggressive genomic signatures [9]. “Infiltrative pattern” was associated with the signatures involved in cellular proliferation, expression of biliary lineage markers, and vascular invasion [9].

Radiogenomics in HCC have also been studied recently using magnetic resonance imaging (MRI) with Miura and colleagues demonstrating improved disease-free survival (DFS) for patients with hyperintense HCC’s in the hepatobiliary phase on gadolinium-ethoxybenzyl-diethlenetriamine pentaacetic acid-enhanced MRI. Patients with HCC have traditionally shown hypointense lesions on the hepatobiliary phase during this type of MRI, however, Miura et al. revealed that hyperintense lesions offer significantly improved DFS at 3 years (90% *vs.* 54%), although OS was not significantly different [10]. Moreover, gene expression analysis showed that these hyperintense tumors upregulated genes involved in normal hepatic metabolism, suggesting well-differentiated, low-grade malignancy [10]. These findings further suggest the potential of radiogenomics as a less invasive biopsy surrogate. Diffusion-weighted approaches have also been used to identify HCC’s with activating Wingless/Integrated signaling pathway (WNT)/β-catenin mutations, which indicate a more favorable prognosis than HCC’s without this mutation. In 2015, Kitao and colleagues correlated imaging features in HCC with immunohistochemical (IHC) expression of WNT/β-catenin activation. The researchers noted lower median contrast-to-noise and enhancement ratios on diffusion-weight imaging for HCC’s with WNT/β-catenin activation, compared to those without it [11]. Furthermore, on IHC analysis, tumors with WNT/β-catenin signaling activation were more likely to be well-differentiated.

Multiparametric magnetic resonance imaging using diffusion-weighted imaging, blood oxygenation level dependent, and tissue oxygenation level dependent methods has also been used in HCC to assess tumor heterogeneity. In a study of 32 HCC patients, data gathered from these multiparametric MRI methods were correlated with histopathology and gene expression. The investigators found that poor tumor perfusion, as measured by multiparametric methods, correlated with high expression levels of vascular endothelial growth factor A (VEGFA) and immune checkpoints, cluster of differentiation 274 (CD274) and cytotoxic T-lymphocyte-associated-protein 4 (CTLA4) [12].

To date, very few studies have examined correlations between imaging features and tumor genotype in ICC. In a 2015 analysis, Sadot et al. performed texture analysis on the CT images of 25 patients with ICC prior to surgical resection. These quantitative and qualitative imaging phenotypes were then correlated to hypoxia marker data extracted from pre-treatment biopsies, including hypoxia inducible factor-1 alpha (HIF-1α), vascular endothelial growth factor (VEGF), and epidermal growth factor receptor (EGFR). The investigators discovered that the quantitative imaging features correlated significantly with EGFR and VEGF expression levels, while qualitative variables, including “tumor liver difference” and “attenuation heterogeneity”, were correlated with VEGF expression [13], as Segal et al. previously noted [5].

### 2.2. Radiomics in Liver Cancers

#### 2.2.1. Radiomics in HCC

**Figure 2:**
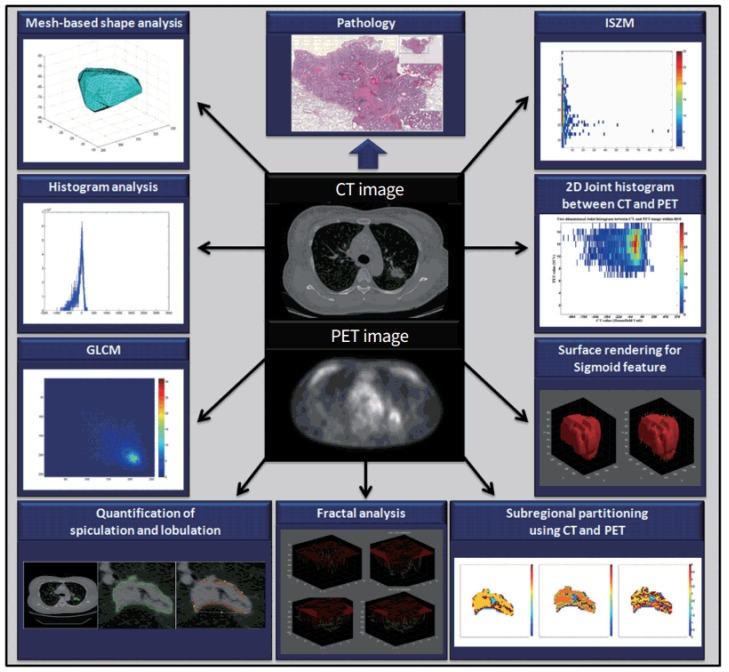
Examples of various radiomics features that can be extracted from CT and positron emission tomography (PET) images. Gray-level co-occurrence matrix (GLCM), histogram analysis, mesh-based shape, intensity size zone matrix (ISZM), two-dimensional joint histogram, surface rendering for sigmoid features, and sub-regional portioning are various features than can be extracted from CT and PET images. These features can then be correlated with clinicopathologic data to gain prognostic and predictive information. Reproduced under a CC-BY-NC 4.0 license from [19].

Radiomics is an emerging field involved with the extraction of high-throughput data from quantitative imaging features and the subsequent combination of this information with clinical data in an attempt to provide prognostic and predictive information from imaging features alone (Figure 2). Several studies have quantitatively analyzed clinical imaging in an attempt to extract prognostic or predictive information in HCC. Kiryu and colleagues assessed the impact of HCC tumor heterogeneity on patient prognosis by analyzing the non-enhanced CT’s of 122 patients undergoing hepatectomy. They identified various texture feature parameters, including the mean, standard deviation, entropy, and mean of the positive pixels, among others, and sought to determine their effects on 5-year progression-free survival (PFS) and overall survival (OS). A number of these texture features were ultimately found to be associated with survival, independent of any effects from other clinicopathologic variables, highlighting the independent prognostic ability of these quantitative imaging features [14]. These results have been mirrored in a number of other studies [15,16,17] that used various methodologies to segment tumors or correlate imaging features with survival. In a study of 38 patients, Xia et al. used intra-tumor partitioning of contrast-enhanced CT scans and available gene expression profiles to identify eight quantitative imaging features that were significantly correlated with prognostic gene modules in HCC. By partitioning tumors into “distinct subregions”, two imaging features were found to be associated with OS [15]. Another study used texture analysis to identify 96 histogram based texture features in 127 resectable HCC patients. These features were then used to train a random survival forest (RSF) classifier to predict DFS and OS [16]. The RSF was able to stratify patients into two groups based upon risk, and ultimately this risk classification, along with vascular invasion, was found to be associated with OS [16].

CT-based radiomics have also been used to preoperatively predict early recurrence in HCC. In a retrospective analysis of 215 patients undergoing partial hepatectomy, Zhou et al. found 21 imaging features to be significantly associated with early recurrence. The predictive performance of this radiomics signature was then compared to that of a model composed of only HCC clinical risk factors, including age, Hepatitis B or C viral status, and Child-Pugh grade, among others. The investigators discovered that the diagnostic performance of the combined radiomics signature and clinical model were superior at estimating early recurrence than either method alone, with a sensitivity of 82.4% and specificity of 70.8% [18]. These results underscore the prognostic ability of these imaging features and also their potential to help stratify patients for treatment selection.

The prognostic utility of radiomics in HCC has also been demonstrated in metabolic imaging using 18-Fluoro-deoxyglucose positron emission tomography (FDG-PET). Previous studies have correlated high FDG uptake with a high grade in HCC [20,21] and more recently, whole-liver radiomics have been used to create a scoring system to predict PFS and OS in unresectable HCC patients undergoing Yttrium-90 radioembolization. In a retrospective cohort of 47 patients, Blanc-Durand and colleagues created a predictive scoring system using 39 imaging features that classified HCC patients into low- and high-risk subgroups [22]. These subgroup classifications were significantly associated with PFS and OS. Furthermore, by incorporating whole-liver radiomics (both tumoral and cirrhotic liver segmentation), the researchers suggested their model incorporated metabolic liver function in addition to tumor biology [22], which has been shown to influence HCC prognosis.

#### 2.2.2. Radiomics in ICC

The radiomics of ICC are not as well-studied as those of HCC; nonetheless, several studies have reported on the prognostic role of imaging features in ICC. In 2011, Kim and colleagues demonstrated that arterially enhancing ICC’s demonstrated less acellular areas and necrosis, and that the arterial enhancement of more than 50% of a given tumor on CT was associated with improved DFS after surgical resection when compared to tumors with less enhancement [23]. These findings were later confirmed in a separate cohort of 47 ICC patients who were grouped into hypovascular, rim-enhancing, and hypervascular subgroups. Those patients with hypovascular tumors were noted to have significantly more instances of lymphatic invasion, perineural invasion, biliary invasion, and, more importantly, poorer DFS when compared to patients in other subgroups [24]. A more recent analysis by Aherne et al. of 66 patients with resectable ICC demonstrated that findings of necrosis or vascular encasement on CT were associated with decreased OS, while the presence of satellite nodules or a larger tumor size were associated with decreased PFS [25]. Interestingly, this study also collected genetic mutation data on these cases; however, no association was found between ICC imaging features and these mutations. The prognostic value of imaging features in ICC has also been investigated on diffusion-weighted imaging. In a cohort of 91 patients grouped into tumors with less than one-third of diffusion restriction and those with greater than one-third diffusion restriction, Lee et al. showed significantly better 1- and 3-year DFS and OS in the group of patients with less than one-third diffusion restriction [26].

#### 2.2.3. Radiomics in Liver Metastases

The prognostic and predictive value of radiomics in colorectal cancer metastases to the liver have been well studied, with several studies demonstrating the utility of diagnostic imaging in predicting clinical outcomes. In an analysis of 77 contrast-enhanced pre-treatment CT scans of colorectal cancer patients, Lubner et al. investigated tumor heterogeneity through analysis of several quantitative texture parameters, including entropy, skewness, mean positive pixels, and standard deviation [27]. Correlations between these imaging parameters and patients’ clinicopathologic features demonstrated an association between less entropy, standard deviation, and a higher mean positive pixel value with tumor grade. Furthermore, the degree of skewness was negatively correlated with KRAS mutations, and entropy was associated with OS [27]. These results were replicated in a retrospective study of 198 preoperative CT scans for patients undergoing resection of colorectal liver metastases. In addition to showing a survival benefit for certain textural imaging features, the investigators were also able to stratify patients by risk of recurrence in the future liver remnant (FLR) based off of imaging features alone. It was found that patients with a more homogenous FLR parenchyma had twice the risk of intrahepatic recurrence compared to patients with less homogenous parenchyma [28]. These imaging biomarkers could potentially be used to stratify patients with a higher risk of local recurrence for a more in-depth follow up. Recently, the radiomics of colorectal metastases to the liver have been studied through MRI on mouse models. After injecting the experimental group of mice with colorectal cancer cells, the researchers used a T2-weighted spin-echo sequence to serially monitor for changes in 32 texture features and development of visible tumor. Visible tumor was appreciated a median of 20 days after injection and three imaging features identified by texture analysis were found to be correlated with metastasis. Changes in these features could be detected by texture analysis prior to any morphologically visible lesion, highlighting the ability of MRI to detect early metastatic lesions not readily apparent to the naked eye [29].

Radiomics analysis has also demonstrated its potential in assessing treatment response for patients with colorectal liver metastases. In a study of 21 patients with pre- and post-chemotherapy CT scans, Rao et al. compared texture analysis to Response Evaluation Criteria in Solid Tumors (RECIST) for assessing response to chemotherapy. Interestingly, they found that quantitative changes in entropy and uniformity were better at differentiating between good and poor responses when compared to changes in size or volume when using the RECIST criteria [30]. These results suggest that texture analysis may be better at predicting treatment response when compared to conventional size criteria, further expanding the utility of diagnostic imaging. MRI-based texture analysis has also been used to analyze treatment response to Yttrium-90 radioembolization in patients with liver metastases. In a retrospective cohort of 37 patients who underwent radioembolization, treatment response was monitored by serial imaging based upon texture analysis and RECIST criteria. The researchers showed that in patients with progressive disease, texture analysis was able to detect progression an average of 3.5 months before RECIST [31]. These findings, in addition to the ones previously addressed, demonstrate the diagnostic, prognostic, and therapeutic implications of imaging features, emphasizing their potential to significantly impact liver cancer outcomes.

## 3. Conclusions

The rapidly developing fields of radiogenomics and radiomics exemplify exciting developments in, and the promising future of, personalized cancer care. Through the correlation of quantitative and qualitative imaging features with genetic data, and the extrapolation of prognostic and predictive information from clinical imaging, radiologists will one day be able to potentially diagnose and stratify patients for treatment based upon imaging features alone. Furthermore, serial monitoring of these radiogenomic and radiomic biomarkers will better allow clinicians to monitor disease recurrence and treatment response, while helping to tailor targeted-therapies to the ever-evolving tumor genome. The results of these studies in liver cancers warrant further investigation into the applicability of radiogenomics and radiomics analyses for a variety of others cancers.
